# Predictive radiomics based ensemble machine learning approach in CT lung nodule diagnosis

**DOI:** 10.1186/s43046-025-00326-7

**Published:** 2025-10-13

**Authors:** Arooj Nissar, A. H. Mir

**Affiliations:** 1 Department of Information Technology, National Institute of Technology Srinagar, Srinagar, 190006 India; 2 Department of Electronics and Communication, National Institute of Technology Srinagar, Srinagar, 190006 India

**Keywords:** Lung cancer, LIDC, Radiomics, Computed tomography, Machine learning

## Abstract

**Background:**

Computed tomography imaging, a non-invasive tool, is used around the globe by medical professionals to identify and diagnose lung cancer; a lethal disease with high rates of occurrence and mortality globally. Radiomics extracted from medical images, including computed tomography, in tandem with machine learning frameworks has received considerable focus and research for lung nodule identification.This investigation can help out clinicians to reach radiomics-based better and quicker decision support system for treatments and early diagnosis. However, it is still foggy and unclear which radiomics feature(s) to use for the prediction of pulmonary nodule. Consequently, this work is offered with an endeavor to efficiently apply machine learning techniques and radiomics to classify CT pulmonary nodules.

**Methods:**

Lung Image Data Consortium (LIDC), containing 1018 CT cancer cases, is put to use. The Wavelet Packet Transform is used in conjunction with geometrical features, gray level run length matrix, gray level co-occurrence method and gray level difference method techniques to extract radiomics. Two techniques, boosted and bagged ensemble classification trees, are employed to choose an apposite set of features. The categorization of nodules as malignant or benign is assessed by the utilization of cutting-edge machine learning models: Support Vector Machines, Boosted Classification Ensemble Tree, Decision Trees, Bagged Classification Ensemble Tree, RUSBoosted Ensemble Trees, Subspace Discriminant Ensemble and Subspace KNN Ensemble.

**Results:**

The findings reveal that the Ensemble Subspace KNN gives best AUROC (93.4%), accuracy (88.3%) and F1-score (85.2%) using BACET feature selection method. The best sensitivity is produced by FGSVM (97.1%). RUSBOCET gives best precision and specificity of 93.4% and 83.1% respectively.

**Conclusion:**

Lung Cancer remains the most common and deadly type of cancer. Early detection of lung lesions and nodules is crucial in the fight against lung cancer. The purpose of this study was to investigate radiomics based on geometrical, texture, and Daubechies WPT texture features for quantitative CT image analysis. The LIDC database was used in this study. Geometrical features, texture features based on three statistical methodologies (GLCM, GLDM GLRLM) and Daubechies WPT texture features are retrieved from the nodules. Using the ensemble EFS, BOCET and BACET, pertinent features were identified. Lastly, various cutting-edge ML classifiers were used to classify LC as malignant or benign. The out-turn shows that, using BACET EFS, Ensemble Subspace KNN gives best AUROC (93.4%), accuracy (88.3%) and F1-score (85.2%). FGSVM yields the best sensitivity of 97.1%. RUSBOCET gives best precision and best specificity of 93.4% and 83.1% respectively. Therefore, the methodology can be applied with efficacy to the CT based PN classification. Thus, the result can assist medical professionals in making better decisions and interventions.

## Introduction

Worldwide, lung cancer (LC) is a serious and prevalent disease that affects both men and women. With 2.21 million instances, it currently holds the second-highest place among all cancer types, and its incidence is steadily rising. The main causes of LC include a variety of factors, including drug intake, smoking and breathing in dangerous pollutants released by automobiles and industry [1]. People over 70 years of age are most affected by LC, with just a small percentage of those diagnosed with this condition being younger than 45 [2]. As per the World Health Organization (WHO) estimate, LC alone results in 1.80 million deaths annually [3]. A study on USA statistics from 2020 states that there were 136,084 deaths from LC and 197,453 new reported cases of LC.Approximately 44,500 cases of LC are detected in the UK each year [4].

Early cancer detection is crucial to reducing the aforementioned statistics. Pulmonary nodules (PNs) are the main focus for such LC diagnosis since they give a clear image of cancer spread. A circular lesion ≤ 3 cm in diameter is what makes up a PN. It could be malignant, which indicates cancerous, or benign, which indicates non-cancerous [2]. Malignant lung nodules significantly increase the risk of death, but if the nodules are found early enough to control the disease, the patient has a higher chance of survival. So, it's necessary to correctly distinguish between benign and malignant nodules in order to get an early and precise diagnosis of LC [5].

The lack of symptoms in the early stages of LC is one of the crucial impediments to its discovery and diagnosis. Many of the instances are known about or found by clinicians when LC reaches an advanced stage, at which point it becomes extremely difficult to cure the illness. Various clinical methods, including radiography, blood testing, endoscopy, biopsies, X-ray imaging, etc. can be used to identify LC. Among these, the non-invasive computed tomography (CT) technology is widely used to diagnose LC because it yields detailed information about the location, size, shape, and other characteristics of the tumor quickly and painlessly [4]. These CT measures, however, are useful but only provide subjective analysis, and because radiologists must manually evaluate the results, there is a significant chance that human error may occur [6].

In recent times, radiomics has become a key focus in LC research. The process of transforming biomedical images into high-dimensional and mineable data is called radiomics. This process is driven by the idea that these images contain crucial information about underlying pathophysiology. These interrelationships can be unveiled using mathematical and machine learning (ML) techniques to dig into the potential connections with biological and clinical outcomes [7]. These imperceptible features have the power to ease clinicians' workload and enhance the process of diagnosis and prognosis. The main objective of radiomics is to create dependable and efficient clinical decision support systems that will aid clinicians rather than supersede them [8]. The diagnosis of disease has changed as a result of the adoption of ML in healthcare. ML algorithms have enhanced potential of handling diverse data kinds and generate highly accurate categorization results. Accurate diagnosis of LC can be achieved by combining radiomics and advanced learning techniques. For LC identification, Noor Khehrah et al. [6] suggested employing shape and statistical attributes in conjunction with Support Vector Machines (SVM). They got good findings, with a 93.75% sensitivity. SVM was used by Parmatasari et al. [9], in another study to categorize LC, and the results exhibited an accuracy of 85.63%.

In radiomics, characteristic attributes are extracted from either 3D Voxels of Interest (VOIs) or 2D Region of Interests (ROIs). The motive of this proposed study is to appraise the proficiency of ML and 2D CT radiomics in lung nodule cancer prediction and diagnosis. The method selects the features that are best suited for classification. Along with SVM, a number of cutting-edge ML classifiers are assessed utilising metrics to determine the best model or outcome. The suggested paradigm is useful and promising for accurately classifying lung nodules as malignant or benign.

### Existing literature

Using CT images, Alzubaidi et al. [4] created a through and comparative framework that addresses both global and local aspects of LC diagnosis. 1000 CT scans were considered. The framework consists of training and testing of global and local features. Six ML algorithm detection models use the feature vectors generated from the features extracted globally. SVM performs better than other learning strategies, whereas the Histogram of Oriented Gradients (HOG), Haar Wavelet feature and Gabor Filter perform better than other features. In comparison to global techniques, Gabor Filter features combined with SVM yield 97% accuracy, 96% sensitivity, and 97% specificity. Chen et al. [5] employed radiomics in the diagnosis, prognosis, and prediction of treatment response. Radiomics and CT images were utilized to classify lung nodules using a 4-feature signature. Malignant and benign nodules varied in 76 out of 750 imaging characteristics in 72 individuals with 75 PNs. The radiomics signature demonstrated an 84% accuracy rate, 92.85% sensitivity, and 72.73% specificity in classifying benign or malignant nodules. According to the study, radiomics can improve noninvasive lung nodule classification. Khehrah et al. [6] automated the identification of nodules using lung CT scans. Statistical, shape-based attributes of suspected nodules, generated to get feature space, are classified by SVM. The methodology's sensitivity is reported as 93.75%. The framework enhances the diagnosis and identification of lung nodules. SVM classification using Gray Level Co-occurrence Method (GLCM) and Gray Level Run Length Method (GLRLM) features is employed to determine LC by Permatasari et al. [9]. In this study, 500 CT scans are divided into LC and normal clusters using data from the Cancer Imaging Archive Database. The study looked into preprocessing, ROI delineation and feature extraction. The obtained SVM classification accuracy is 85.63%. As a way to classify CT PNs, Donga et al. [10] looked into modified gradient-boosting ML. To distinguish between benign and malignant nodules, the preprocessing of CT scans, ROI segmentation, extracting texture as well as intensity data, then training and evaluation the model classifier is done. The proposed framework shows good precision, recall, F1-score, and validation accuracy (0.957%, 0.91, 0.941, and 95.67%) on the Lung Image Data Consortium (LIDC) dataset. Comparative studies reveal that the recommended method diagnoses lung nodules better.

Shakir et al. [11] used CT scans to create radiomics-driven models for the classification of colon, lung, and neck-head cancer. Out of 105 3-D characteristics, analytical radiomic signatures were extracted. Analysis based on regression was performed using these signatures to classify tumors. High classification rates on a public dataset of 265 images showed that the models were robust throughout validation. According to the study, general tumor phenotype-based mathematical diagnostic functions for cancer diagnosis have been successfully developed. Palumbo et al. [12] evaluated the effectiveness of 18F-FDG PET/CT-based shape and texture features to differentiate between benign and malignant lung nodules. Prediction models used eighteen (3D) features from Positron Emission Tomography (PET) and CT. The model results unequivocally demonstrate that combining PET and CT characteristics results in an additional accuracy gain. The longevity of CT scan radiomics features for Non-Small Cell Lung Cancer (NSCLC) in relation to three segmentation procedures was investigated by Belfiore et al. [13]. Three 3D-ROIs were segmented by skilled radiologists in order to examine radiomics features in 48 NSCLC patients. An intra-class correlation coefficient (ICC) was computed for the characteristics. Shape attributes showed minimal parameter sensitivity and strong agreement (ICC > 0.9). There was good agreement between a subset of first-order and second-order attributes. The study discovered that NSCLC CT scan repeatability can be greatly increased by specific radiomics features. In low-income countries lacking lung biopsies, Padmakumari et al. [14] investigated the efficacy of CT radiomics in differentiating between LC and tuberculosis (TB). From 3D segmented CT scans of the chests of individuals with histologically confirmed TB or LC, radiomics were generated. LC and TB differed significantly in terms of radiomics and clinical characteristics. The study demonstrated how non-invasive radiomics diagnosis of cancer patients may improve treatment for those with minimal resources. Further research is required to confirm these results, though. The work in [15] created a radiomics nomogram to distinguish between benign and malignant lung tumors based on wavelet features. Out of 116 patients, training and validation sets, *N* = 70 and *N* = 46 respectively, were considered having solitary PNs (SPNs) of size 3 cm. The radiomics parameters of each patient were extracted using standard CT images. The researchers produced a radiomics nomogram in the training set with a 95% accuracy and an area under the curve (AUC) of 0.9406, using a multivariate logistic regression model. Moreover, the validation set's AUC was 0.8454 with a 95% accuracy rate.

A hybrid technique combining feed forward networks with nodule radiomics from CT scans was tested by Torres et al. [16]. To increase repeatability with less training data, they proposed integrating statistically significant radiomic characteristics for malignancy detection. The optimal model in a separate patient population detected cancers with 83% specificity, 100% sensitivity and AUC 94%. In a separate investigation, Balci et al. [17] presented a novel hybrid methodology that uses radial scanning series features along with medical image analysis to perform classifications. 92.84% accuracy, 92.41% recall, and 92.63% precision were attained, according to the findings. Most recent reviews, based on the budding future of radiomics in prediction, prognosis and treatment of LC, are reported in [18–21].

From the above studies and many more it is established that not every extracted feature is a credible predictor for lung nodule classification. Therefore, it is intriguing and crucial to identify the most discriminative features, or a subset of them, from an extracted feature pool to facilitate the development of a fast and more reliable clinical decision support system. This study attempts to investigate and analyze the discriminative potential of several geometrical characteristics, statistical texture features and WPT texture features. Using feature selection (FS) techniques, the most important and discriminative characteristics are selected from a large pool of 7455 retrieved features. The performance of each diagnostic model is assessed by applying cutting-edge ML classification techniques to the relevant features.

The remainder of the paper goes like this: Section "Methods" presents the methods. Section "Results and discussion" contains findings of the experiment and the discussion. The conclusion and future work are covered in Section "Conclusion and future work".

## Methods

The motive of this research project is to determine whether nodules in CT lung scans are benign or malignant by examining the effectiveness of geometrical and 2D radiomics, FS techniques and ML algorithms. The whole study framework comprises of various phases, viz. Collection of dataset, feature extraction, feature selection, classification and assessment of various classifiers.

### Dataset

**Fig. 1:**
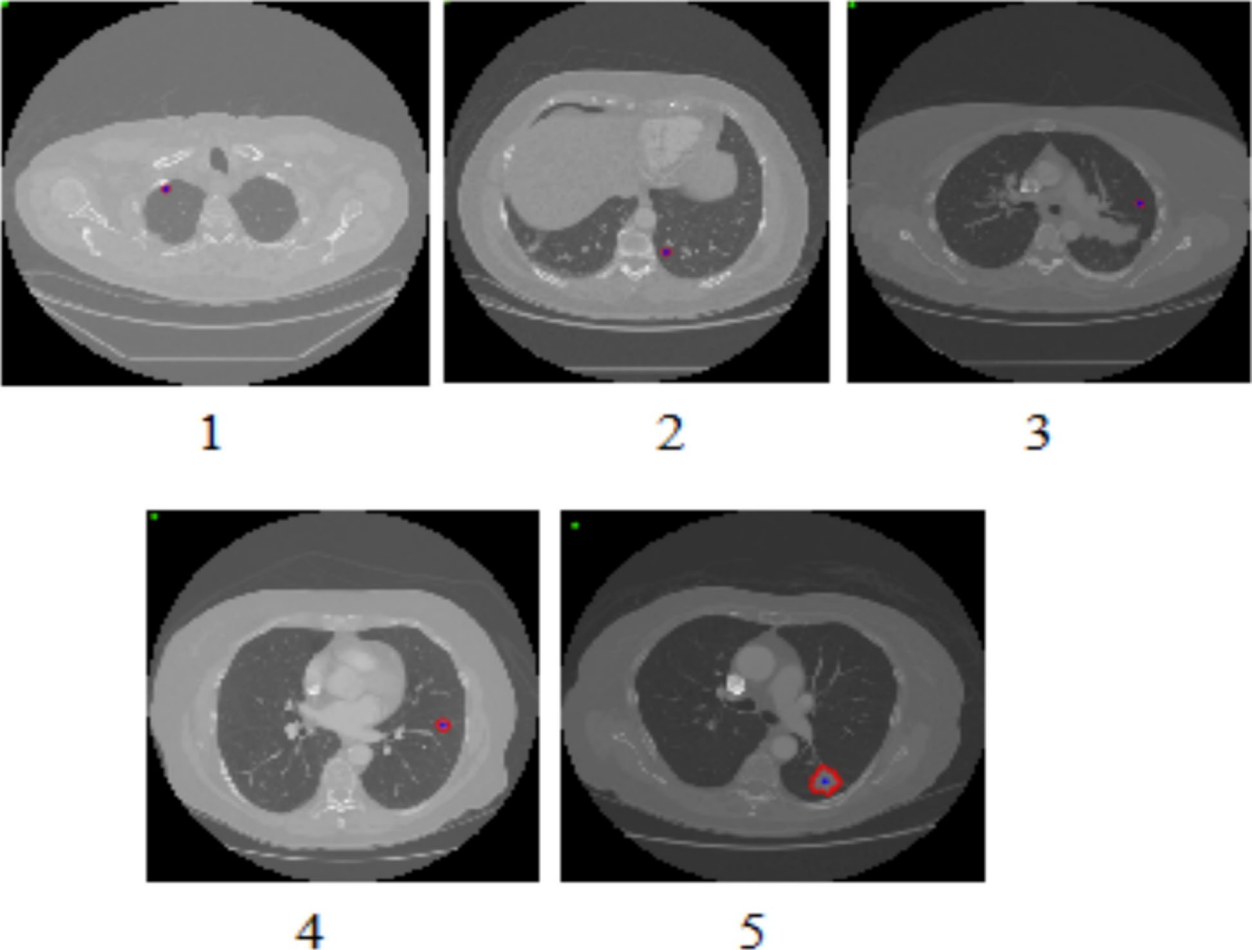
LIDC dataset sample images with a malignant ROIs

Any diagnostic system relies substantially on database. This study makes use of CT images from LiDC, which has a collection of 1018 CT LC patient scans. This LIDC database contains both CT images and ground truth reports from four experienced radiologists. McNitt-Gray et al., [22–24] provide a detailed description of the existence of malignancy in nodules ≤ 3 cm and radiologists' remarks. The total count of the slices per CT used in this investigation ranged between 110 to 388. A total of 1207 CT scan slices were considered, including 883 malignant and 324 benign. Figure 1 shows sample CT images from the LIDC dataset that contain malignant ROIs.

### Feature extraction

The aforementioned dataset was used to extract features. Statistical approaches are used to extract radiomics (geometrical, texture and wavelet). Here's a synopsis of these features.

#### Geometrical features

Disparate geometrical attributes play a significant part in the classification procedure. These attributes are imperative since they have a direct bearing on the diagnosis and prognosis of cancer [25]. The 7 attributes viz. Minor axis Length, Major axisLength, Mean-Intensity, Max-Intensity, Min-Intensity Area and Perimeter are computed. Table 1 presents a list of these features.

**Table 1:** List of geometrical and texture features extracted using GLCM, GLDM and GLRLM [26]

GLCM	Auto-correlation (ACOR), Contrast (CON), Correlation1(COR1), Correlation2 (COR2), Cluster Prominence (CP), Cluster Shade (CS),Dissimilarity (DS), Energy (ENR), Entropy (ENT), Homogeneity1(HMG1), Homogeneity2 (HMG2), Maximum Probability (MP), Sum of Squares: Variance (SOS), Sum Average (SA), Sum Variance (SV), Sum Entropy (SE), Difference Variance (DV), Difference Entropy (DE), Information Measure of Correlation1 (IMC1), Information Measure of Correlation2 (IMC2), Inverse Difference Moment(IDM), Inverse Difference Moment Normalized (IDMN)
GLDM	Contrast (CON), Angular Second Moment (ASM), Entropy (ENT), Mean, Inverse Difference Moment (IDM)
GLRLM	Short Run Emphasis (SRE), Long Run Emphasis (LRE), Gray Level Non-uniformity (GLN), Run Length Non-uniformity (RLN), Run Percentage (RP), Low Gray-Level Run Emphasis (LGRE), High Gray-Level Run Emphasis (HGRE), Short Run Low Gray-Level Emphasis (SGLGE), Short Run High Gray-Level Emphasis (SRHGE), Long Run Low Gray-Level Emphasis (LRLGE), Long Run High Gray-Level Emphasis (LRHGE)
Geometric Features	Area, Perimeter, MajorAxisLength, MinorAxisLength, Max_Intensity, Mean_Intensity, Min_Intensity

#### Halarick’s Texture features

Texture analysis facilitates the interpretation of the heterogeneity of tissues, a trait thought to affect how well cancer diagnosis succeeds [27]. It does this by capturing the spatial distribution of intensities [28]. Texture features are investigated using three techniques: GLCM, GLRLM and Gray Level Difference Method (GLDM) [6, 11, 12, 26, 29]. Second and high-order statistics are extracted based on inter-pixel distance 'd' and angle 'θ'. 22 texture features are calculated with GLCM: Auto-correlation (ACOR), Correlation2 (COR2), Correlation1 (COR1), Dissimilarity (DS), Cluster Prominence (CP), Energy (ENR), Entropy (ENT), Cluster Shade (CS), Maximum Probability (MP), Homogeneity1 (HMG1), Homogeneity2 (HMG2), Sum Average (SA), Contrast (CON), Information Measure of Correlation2 (IMC2), Information Measure of Correlation1 (IMC1), Inverse Difference Moment (IDM), Difference Variance (DV), Sum of Squares: Variance (SOS), Sum Variance (SV), Difference Entropy (DENT), Sum Entropy (SENT), Inverse Difference Moment Normalised (IDMN). Refer to Table 1.

GLDM calculates 5 features: Entropy (ENT), Contrast (CON), Mean (M), Inverse Difference Moment (IDM) and Angular Second Moment (ASM). Refer to Table 1.

In addition, 11 features are computed using GLRLM: Short Run Low Gray-Level Emphasis (SGLGE), Short Run Emphasis (SRE), Short Run High Gray-Level Emphasis (SRHGE), Gray Level Non-uniformity (GLN), Run Length Non-uniformity (RLN), Run Percentage (RP), Low Gray-Level Run Emphasis (LGRE), High Gray-Level Run Emphasis (HGRE), Long Run Emphasis (LRE), Long Run Low Gray-Level Emphasis (LRLGE) and Long Run High Gray-Level Emphasis (LRHGE). See Table 1.

#### WPT—Texture features

2-level Wavelet Packet Transform (WPT) [30, 31] is used to create multi-scale interpretations of the actual image. Utilizing wavelet-based features offers a multitude of benefits, including their exceptional ability to capture even the most intricate details, while remaining resilient to noise and variability in imaging conditions. Although there are many well performing wavelets available, the type of wavelet employed is application dependent. The orthogonal wavelets of compact support that Daubechies [32] developed were the main subject of this work. These wavelets can have a significant impact on how well texture analysis and classification work as the filter raises the standard of the identifiers [2, 33]. To implement WPT, the Daubechies wavelet family: db1, db2, and db3 was utilized. This 2-level WPT produces 16 multi scaled images given one image. These images are used to compute the attributes listed above (Section "Halarick’s Texture features"). These classes are therefore denoted as WPT-GLCM, WPT-GLDM, and WPT-GLRLM. Table 2 provides a list of feature classes and the total number of features extracted for each class. The list of extracted attributes is presented in Table 1.

**Table 2:** List of features per class

Feature class		No of the features extracted				Total
Geometric		7				
GLCM	Directions (θ)	*0*^*0*^	22	88	152	7455
		*45*^*0*^	22			
		*90*^*0*^	22			
		*135*^*0*^	22			
GLDM		*0*^*0*^	5	20		
		*45*^*0*^	5			
		*90*^*0*^	5			
		*135*^*0*^	5			
GLRLM		*0*^*0*^	11	44		
		*45*^*0*^	11			
		*90*^*0*^	11			
		*135*^*0*^	11			
WPT-GLCM	WPT family (Level = 2)	*db1*	88*16	1408	4224	
		*db2*	88*16	1408		
		*db3*	88*16	1408		
WPT-GLDM		*db1*	20*16	320	960	
		*db2*	20*16	320		
		*db3*	20*16	320		
WPT-GLRLM		*db1*	44*16	704	2112	
		*db2*	44*16	704		
		*db3*	44*16	704		

### Feature selection

FS or reduction aims to extract only the most beneficial attributes and eliminate noisy, unwarranted, and duplicitous attributes in order to benefit ML models [34]. This FS is the most crucial step in getting ready for ML model training. This is done as a way to avoid over-fitting, which may boost the accuracy of model forecasts and its capacity to generalize while also aiding in the construction of a strong radiomic signature [20]. The routinely used FS methods are put into three methodological classes: filter, wrapper and embedded methods. In present work two embedded FS methods (EFS) were employed viz. Boosted Classification Ensemble Tree (BOCET) and Bagged Classification Ensemble Tree (BACET).

The EFS stands out as a powerful technique that strikes a perfect balance between filters and wrappers. By handpicking features that surface during the learning process and aligning them with the classifier's evaluation criteria, this method drastically reduces computational expenses compared to wrappers. EFS entail integrating the process of FS directly into the model training process and help to reduce the time needed to reclassify subsets [35]. In order to attain optimal classification accuracy, the classifier modifies its internal parameters and determines the right weights or priorities assigned to each feature throughout the training process. One example of EFS techniques includes algorithms based on decision tree like gradient boosting, random forest and decision tree. Another effective EFS method is FS using regularization models such as LASSO. When used with linear classifiers like logistic regression and SVM, regularization algorithms typically work by penalizing the coefficient of features that do not substantially enhance the model performance [36]. It is worth noting that the previously stated regularization and decision tree based techniques yield a ranked list of characteristics. A tree ensemble is a bagging algorithm that combines a set number of decision trees. The tree based tactics naturally rank by how well they improve the node purity or reduce impurity (Gini impurity) across all trees. The nodes at the beginning of the trees have a greater decrease in impurity, while the nodes at the end of the trees have the least amount of impurity decrease. Thus, by pruning trees below a specific node, we can produce a subset of the most essential attributes.

**Fig. 2:**
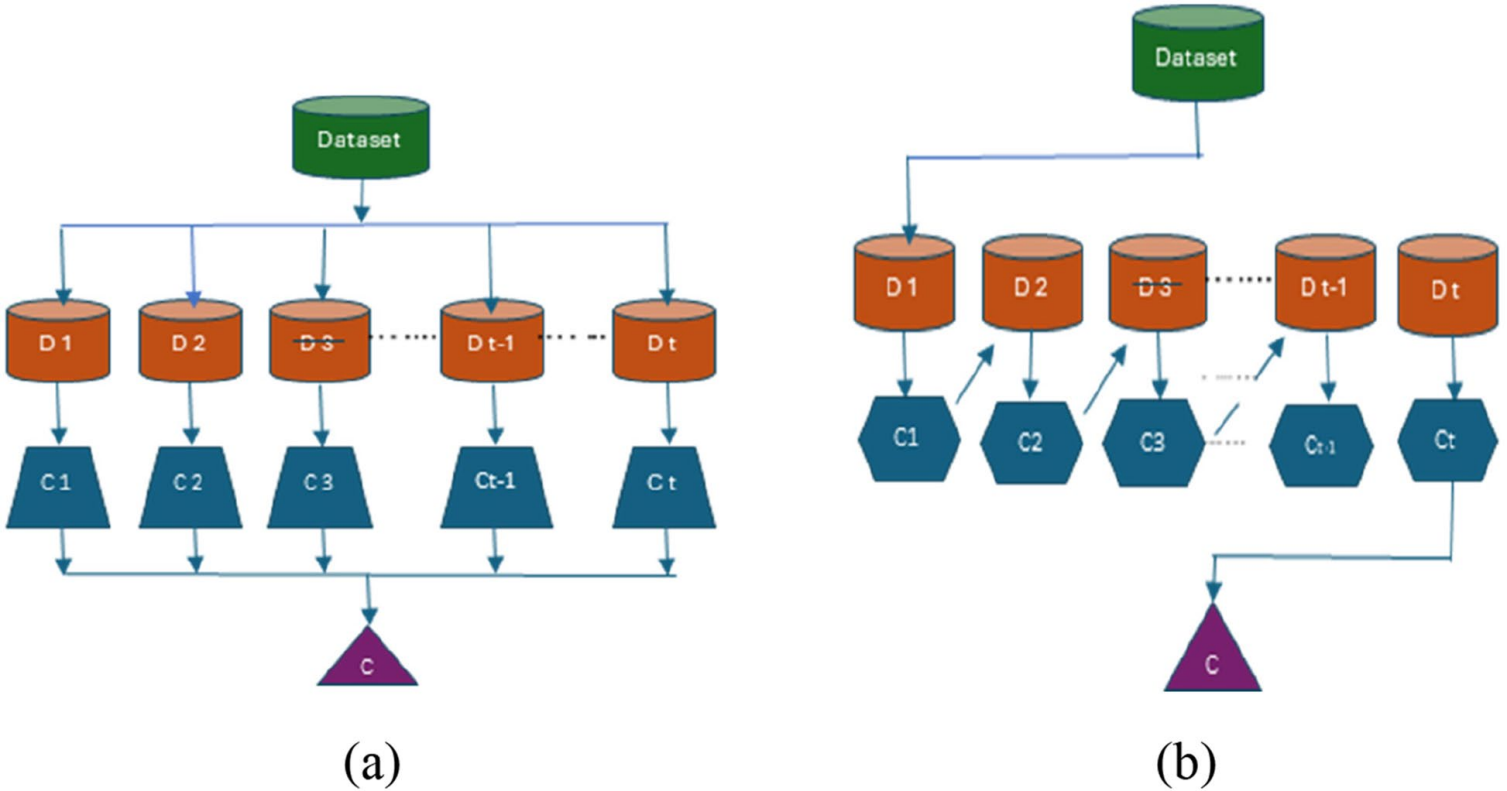
**a** Bagging and **b** boosting

Two different types of ensemble learning methods, bagging and boosting, are used for EFS. BOCET [37] and BACET [38] both approaches dynamically detect and prioritize pertinent features while constructing intricate models. Illustration of BACET and BOCET is given in Fig. 2(a) and (b). The algorithms for both are presented in Algorithm 1 and Algorithm 2.

**Figure:**
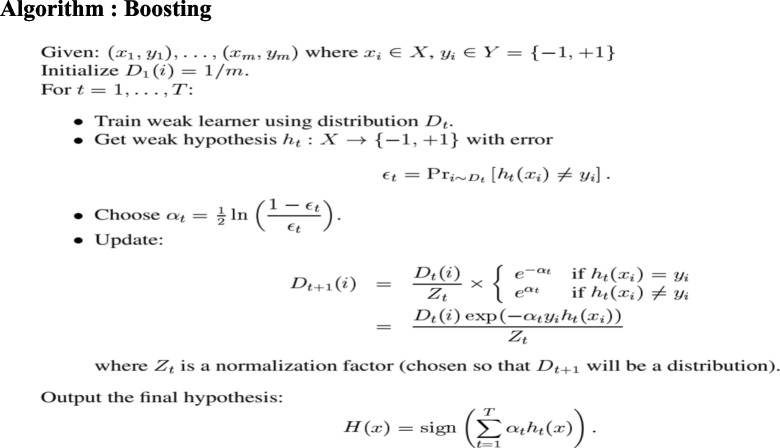
**Algorithm 1** Pseudocode for the boosting algorithm AdaBoost

**Figure:**
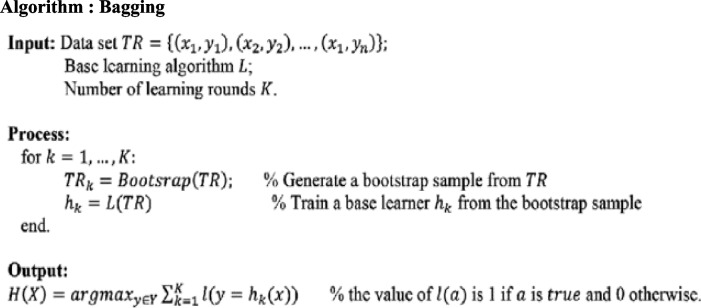
**Algorithm 2** Pseudocode for the bagging algorithm

### Categorization and performance evaluation

#### Classification

Using EFS algorithms, when discriminative radiomics are obtained, the lung nodules can be classified into two classes (benign and malignant) using a number of cutting-edge ML classifiers, including SVM [39–41], decision trees (DT) [42], Ensemble Trees [43, 44], and Ensemble Subspace [45, 46].

*SVM*: According to research, SVM's relative simplicity and adaptability in implementation have rendered it as a powerful and effective ML strategy in the field of biomedical image analysis [40]. SVM can handle non-linearly independent data by using a kernel function to transform the input attributes into a higher-dimensional space. The various kernel functions that are utilized are sigmoid, polynomial, linear and Radial Basis Function (RBF, often known as Gaussian).

*DT*: A tree structure is used in the classification process using the DT algorithm in machine learning. The dataset, the root node, is broken down into nodes using this approach. Every internal node represents a feature, branches stand for rules, and leaf nodes represent classification and decision-making. One can apply this strategy to both qualitative and numerical data.

*Ensemble Tree*: An ensemble tree is a supervised ML technique that comprises of an ensemble of independently trained DT, which are base learners and might not work well when used singly. A new strong model is created by aggregating the base learners, and this model is often more accurate than the previous ones. Bagging, boosting and RUSboosting are the three types of ensemble tree ML methods used. Bagging creates an assortment of bootstrapped data (bags) from the original training dataset. Each bag contains N observations picked at random from the original dataset with replacement. Thus, a bag consists of around 63% of different samples, with the remainder being duplicates [47]. A DT is then trained with each bag, and the outcomes are aggregated via voting by majority. *Boosting*, an ensemble modeling technique, attempts to construct a robust classifier out of a sequence of weak classifiers. Using the training data, first model is constructed. Next, in an effort to rectify the errors in the first model, a second one is constructed. The aforementioned procedure is iterated until the maximum number of models is added or the full training data set is accurately predicted. An ML approach called RUSboost is used to enhance the performance of models that have been trained on skewed data. Until a desirable class distribution is reached, it uses a technique called random undersampling (RUS), which randomly removes examples from the majority class.

*Ensemble Random Subspace*: In order to reap the benefits, the random subspace (RS) ensemble classifier applies a random portion of features over the combined set of basis classifiers (KNN and Discriminant).The classifier randomly selects a portion of features from the actual dataset and uses them to train some number of weak classifiers. The predicted outputs of these weak classifiers are combined employing a majority voting combination rule to obtain the final target class labels. The non-parametric K-Nearest Neighbor algorithm (KNN) is an instance based classification method. The K training instances that are closest in the feature space make up the input. The input comprises the K closest training examples in the feature space and the output denotes the class membership. A majority vote of neighbors’ establishes the classification. The class is the single closest neighbor if K = 1 [48]. Linear Discriminant Analysis (LDA), a supervised learning method, utilizes class labels, making it ideal for class separation. It employs both within class and between class scatter matrices. When there are two classes, LDA creates a hyperplane and projects the data onto it to increase the distance between the two. This hyperplane is formed by maximizing the difference across the means of two categories and minimizing the variance within each and every category. LDA is commonly used in medical-computer interfaces due to its high accuracy [49].

#### Performance evaluation

Actual and predicted outcomes from prediction models for LC classification are displayed in a confusion matrix. The confusion matrix for LC classification with two outcomes is given in Table 3. True-positive (*tp*) refers to the number of malignant nodules rightly classified as LC. False-positive (*fp*) refers to the number of benign nodules diagnosed as LC. False-negative (*fn*) refers to the number of malignant nodules erroneously classified as benign. True-negative (*tn*) refers to the number of benign nodules classified correctly. The performance evaluation criteria employed in this study include Area under Curve (AUC), Accuracy, Precision, Sensitivity/recall, Specificity, and F1-score.

**Table 3:** Confusion matrix for LC prediction

		Predicted Class	
		NLC 0	LC 1
Actual Class	NLC 0	*tn*	*fp*
	LC 1	*fn*	*tp*

The Area under Curve (AUC) tells how well a model performs.

Accuracy refers to the model's capacity to accurately predict outcomes relative to the entire number of outcomes, as either malignant or benign.

1 $$Accuracy=\frac{tp + tn}{tp + tn + fp + fn}$$

Precision refers to the models’ ability to predict the quality of positive prediction.

2 $$Precision=\frac{ tp}{tp + fp}$$

Sensitivity/Recall is used to compute the number of true positives (*tp*). In the medical field, sensitivity is prioritized over precision as the goal is to identify all true positive cases [50].

3 $$Recall=\frac{tp}{tp + fn}$$

Specificity refers to the ability of the model in predicting true negatives (*tn*).

4 $$Specificity=\frac{tn}{tn + fp}$$

F1-score combines precision and recall into a single measure in a balanced manner.

5 $$F1-score=2\times \frac{Precision \times Recall}{precision +Recall}$$

For all these metrics, a value closer to one signifies an excellent classification result and vice-versa.

**Fig. 3:**
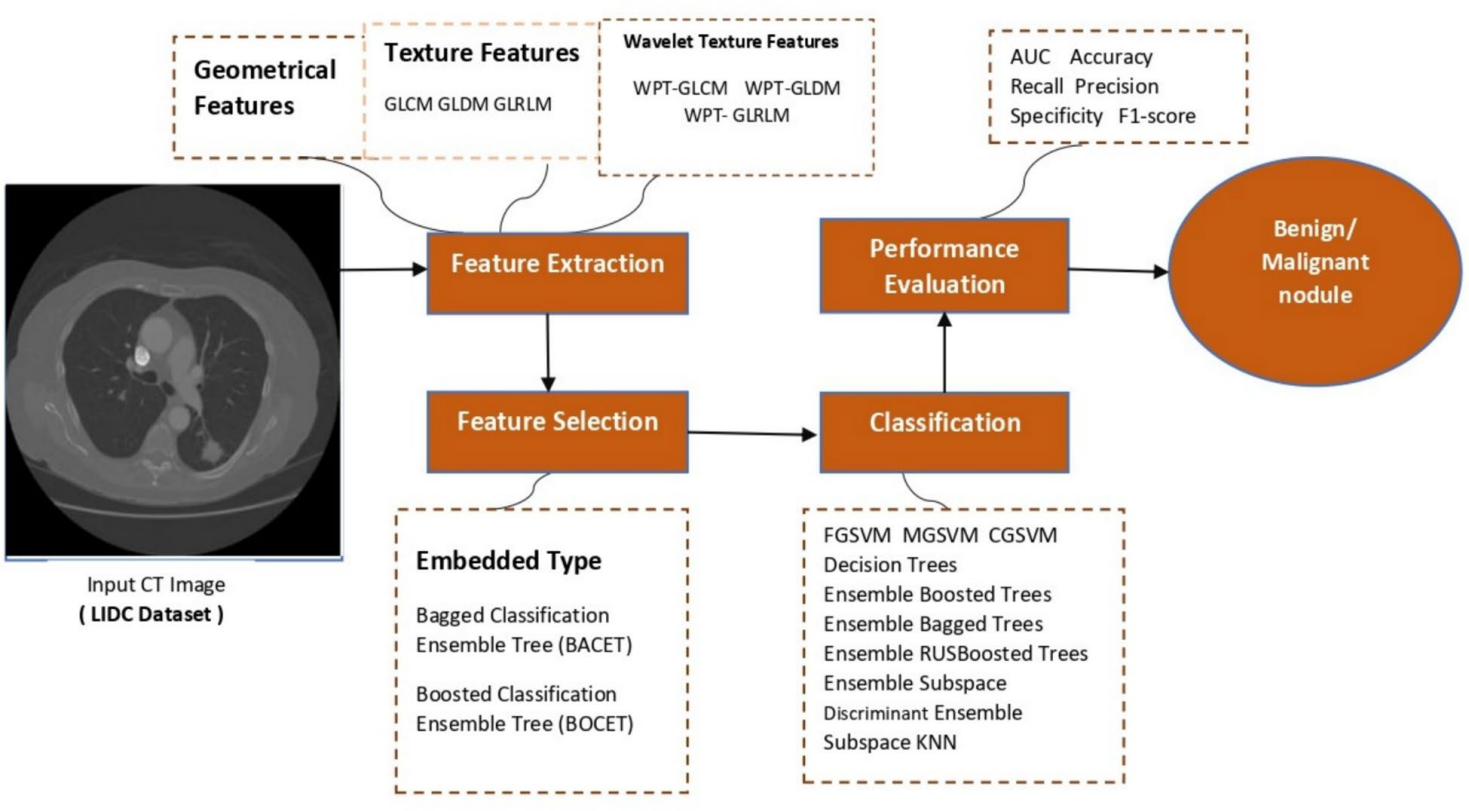
Proposed framework to classify lung nodule as malignant or benign

Figure 3 depicts the framework for the proposed methodology.

## Results and discussion

The proposed strategy was put into practice utilizing MATLAB 2017a and 2021b. For the purpose, a 64-bit with 16 GB RAM computer system was used. The LiDC database is utilized for experimentation. In all 1207 CT scan slices were considered, 324 benign and 883 malignant.

For all slices ROI of nodules were attained using the of the radiologists' annotations. Geometrical features (Section "Geometrical features") are extracted from all nodules. Around the centroid of each nodule ROI, a sub-image of 11 × 11 pixels is chosen. All 152 Haralicks’ texture features (Section "Halarick’s Texture features") are calculated. Four spatial directions at θ = 0°, 45°, 90^o^ and 135° are used to calculate the GLCM, GLDM, and GLRLM matrices, with the interpixel distance ‘d’ = 1. This formulation may have major implications. Additionally, 2-level WPT is applied on each sub-image, yielding 16 multi-scaled minuscule images. The WPT texture features (Section "WPT - Texture features") were assessed in each of the four directions as previously stated, using the Daubechies wavelet family's db1, db2, and db3 as the basis functions. In all, a total of 2112 WPT-GLRLM features, 960 WPT-GLDM features and 4224 WPT-GLCM features were extracted. As a result, 7455 characteristics from the cohort were obtained (Table 2). Feature scaling (min–max normalization) is employed to normalize the range of features for further analysis.

**Fig. 4:**
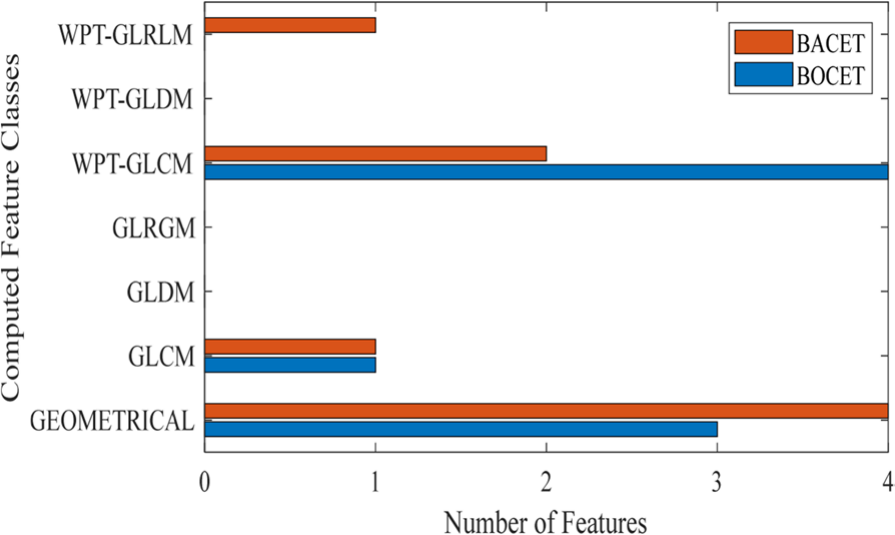
Distribution of top 8 radiomic features w.r.t. feature classes

Two EFS approaches, BOCET and BACET, were used to extract the highly discriminative features from this cohort of 7455 features. To distinguish benign nodules from malignant nodules, the first eight features were chosen based on the ranking presented separately by both approaches. We only used the first eight features for categorization, as adding more features did not result in any further improvement in performance measures. The highly discriminating chosen features for BOCET are: Area, Perimeter, db1_LH_1_HL_2__WPT_GLCM_CP_45^0^, db3_LL_1_LH_2__WPT_GLCM_CP_0^0^, GLCM_CS_0^0^, MajorAxisLength, db1_LL_1_LH_2__ WPT_GLCM_CP_0^0^ and db1_HL_1_LL_2__ WPT_GLCM_CS_0^0^. Whereas the highly discriminating chosen features for BACET are: MajorAxisLength, Area, Perimeter, MinorAxisLength, GLCM_CP_90^0^, db2_LH_1_HH_2__WPT_GLCM_CS_45^0^, db3_LH_1_LH_2__WPT_GLRLM_GLN_0^0^ and db1_HH_1_HH_2__ WPT_GLCM_CP_0^0^. Two sets of discriminative characteristics were available at hand for classification in the subsequent stage (Table 4). Analyzing these selected features it can be seen that Area, Perimeter, MajorAxisLength, CP and CS were ranked as most informative features and hence will play an important part in classification. The distributions of the selected features using above EFS methods w.r.t their feature classes are compared in Fig. 4.

**Table 4:** Listicle of topmost 8 significant features selected by two EFS techniques

Rank	BOCET		BACET	
	*Feature Index*	*Feature Name*	*Feature Index*	*Feature Name*
1	1	Area	1	Area
2	4	Perimeter	4	Perimeter
3	1545	db1_LH_1_HL_2__WPT_GLCM_CP_45^0^	2	Major axis length
4	5344	db3_LL_1_LH_2__WPT_GLCM_CP_0^0^	3	Minor axis length
5	28	GLCM_CS_0^0^	26	GLCM_CP_90^0^
6	2	Major axis length	4285	db2_LH_1_HH_2__WPT_GLCM_CS_45^0^
7	480	db1_LL_1_LH_2__ WPT_GLCM_CP_0^0^	6198	db3_LH_1_LH_2__WPT_GLRLM_GLN_0^0^
8	788	db1_HL_1_LL_2__ WPT_GLCM_CS_0^0^	1646	db1_HH_1_HH_2__ WPT_GLCM_CP_0^0^

**Fig. 5:**
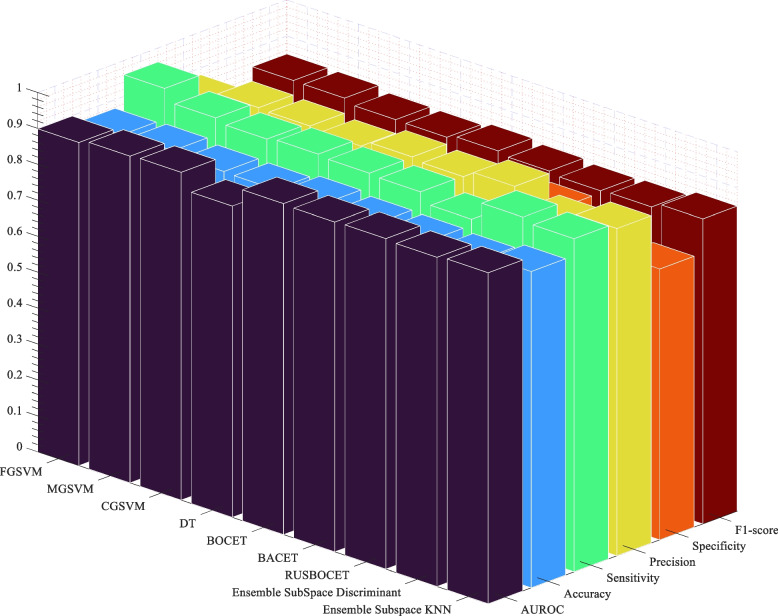
The performance comparison of nine learning methods using BOCET as EFS

**Fig. 6:**
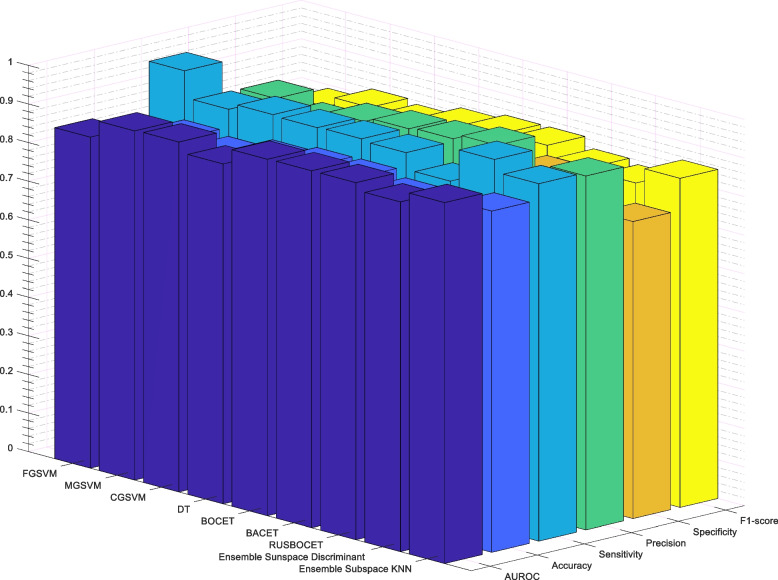
The performance comparison of nine learning methods using BACET as EFS

Several cutting-edge ML classifiers, including DT, BOCET, BACET, Ensemble Subspace Discriminant, Fine Gaussian SVM (FGSVM), Medium Gaussian SVM (MGSVM) and Course Gaussian SVM (CGSVM), Ensemble Subspace KNN and Ensemble Subspace Discriminant (Section "Classification") were assessed and compared in this proposed study. This is done in order to determine the efficacy of two distinct radiomic signatures and, consequently, EFS methods in detecting lung nodules. A fivefold cross-validation strategy was employed and assessed about 50 times in order to provide cross-validated AUC for each classifier in the classification process. All are assessed using a confusion matrix, and the F1-score, precision, sensitivity, accuracy, and AUC are compared. Table 7 offers an in-depth analysis of the aforementioned measures in relation to various classifiers and ranking algorithms. The Table 7 results are also illustrated in Figs. 5 and 6.

In our experiments the selected radiomic features were examined with above 9 ML classifiers to assess the proficiency of radiomic attributes and the classifier duo. The discriminative attributes selected employing each EFS method, BOCET and BACET, give favorable results. As is evident from the Table 7, the performance measures for various ML classifiers have varied values. These variations can be attributed to the deployment of the FS method, the radiomic signature derived, and the ML classifier type. From the metrics acquired from several classifiers, following conclusions can be drawn:

I. BACET EFS method provides the overall best sensitivity/recall (97.1%) with FGSVM followed by Ensemble Subspace Discriminant (95.7%). However, BOCET EFS technique also gives reasonably very good sensitivity with FGSVM (96.1%).
II. The best outcome for AUC (93.4%), accuracy (88.3%) and F1-score (85.2%) are given by Ensemble Subspace KNN model using BACET.
III. Again using BACET FS method, Ensemble RUSBoosted Trees gives highest values for precision (93.4%) and specificity (83.1%).
IV. BACET EFS technique gives better performance results hence proves superior to BOCET.
V. Examining the recall/sensitivity metric, it is evident that all classifiers, with the exception of Ensemble RUSBoosted Trees, are producing promising results.
VI. sensitivity/recall metric it can be spotted that all classifiers are furnishing promising results but Ensemble RUSBoosted Trees.
VII. Furthermore, Ensemble Subspace KNN (BACET) produces results that are generally better for various assessment metrics. [F1-score (85.2%), Sensitivity (92.5%), Specificity (76.9%), Precision (91.6%),AUROC (93.4%), Accuracy (88.3%)]

Taken together, the results presented in Figs. 5 and 6, Tables 4 and 5, we can infer that our results and the techniques (geometrical, CT Daubechies WPT texture features and ML techniques) could be efficiently employed for the cataloguing of PNs into benign or malignant. These frameworks enable comprehensive analysis of nodule features which is done non-invasively and at minimal patient risk. Hence, the boons of using radiomics and ML make cancer diagnosis and prognosis easier, more promising by automating the procedure of FS and classification. It can significantly reduce the possibility of human error while simultaneously saving valuable time in the diagnostic process. It also can lead to more efficient patient care, and ultimately, save lives.

**Table 5:** Classification results attained for nine different State-of-art classifiers using two EFS techniques. (Overall highest values are marked in bold)

***Feature Selection Algorithms***	***Classifiers***	***(Malignant Vs Benign)***					
		***AUROC***	***Accuracy***	***Sensitivity/Recall/TPR***	***Precision/PPV***	***Specificity/TNR***	***F1-score***
*Boosted Classification Ensemble Tree*	FGSVM	0.899	0.876	0.961	0.881	0.646	0.85
	MGSVM	0.909	0.88	0.927	0.911	0.754	0.849
	CGSVM	0.912	0.87	0.916	0.908	0.747	0.837
	Decision Trees	0.866	0.868	0.922	0.9	0.72	0.836
	Ensemble Boosted Trees (BOCET)	0.921	0.879	0.917	0.918	0.775	0.846
	Ensemble Bagged Trees (BACET)	0.918	0.871	0.913	0.911	0.757	0.838
	Ensemble RUSBoosted Trees (RUSBOCET)	0.920	0.869	0.885	0.932	0.825	0.831
	Ensemble Subspace Discriminant	0.916	0.864	0.939	0.882	0.658	0.834
	Ensemble Subspace KNN	0.921	0.88	0.927	0.911	0.754	0.849
*Bagged Classification Ensemble Tree*	FGSVM	0.857	0.813	**0.971**	0.811	0.383	0.791
	MGSVM	0.904	0.856	0.902	0.901	0.729	0.82
	CGSVM	0.906	0.844	0.919	0.874	0.639	0.811
	Decision Tree	0.880	0.866	0.916	0.903	0.732	0.833
	Ensemble Boosted Trees (BOCET)	0.923	0.877	0.918	0.915	0.766	0.845
	Ensemble Bagged Trees (BACET)	0.924	0.878	0.913	0.92	0.781	0.845
	Ensemble RUSBoosted Trees (RUSBOCET)	0.924	0.86	0.87	**0.934**	**0.831**	0.819
	Ensemble Subspace Discriminant	0.905	0.835	0.957	0.84	0.5	0.809
	Ensemble Subspace KNN	**0.934**	**0.883**	0.925	0.916	0.769	**0.852**

### Comparison with previous works

The results of our proposed methodologies are compared and contrasted with previously five published methods [4, 51–54] given in the literature. The results of the comparison are presented in Tables 6 and 7 for BACET and BOCET FS methods respectively. Our proposed classification model 1 and model 10 are found to perform better in terms of sensitivity than all of the remaining models and the previously published methodologies. With respect to precision our proposed classification model 7 and model 16 both outperform above stated previously published methods and all of the remaining models. However with regards to accuracy and specificity best values are reported by Alzubaidi et al*.* [4]. Additionally, Xie et al. [53] and Xie et al. [54] provide the best possible values for the AUROC and F1-score, respectively. The research study presented in [54] computes F1-score, sensitivity, precision, specificity, accuracy and AUC so a full comparison is feasible.

**Table 6:** Comparison of performance metrics of proposed models with previous work when FS method is BOCET

	**Methods**	**# of benign(B) and malignant nodules (M), database**	**Results (%)**					
			**Accuracy**	**Sensitivity**	**Precision**	**Specificity**	**F1-score**	**AUROC**
Dhara et al*.* [51]	2D and 3D GLCM features + SVM	279 B and 263 M, LIDC	-	89.73	-	86.36	-	95.05
Wang et al*. * [52]	Shape + intensity + Gabor + SVM	200 B and 200 M, LIDC	86	82.5	88.7	89.5	-	-
Xie et al. [53]	DCNN + Fourier + UPLBP	1324 B and 648 M, LIDC	88.28	84.15	-	90.12	-	95.70
	DCNN + Fourier + GLCM	1324 B and 648 M, LIDC	89.53	84.19	-	**92.02**	-	**96.65**
Xie et al. [54]	Multi-view knowledge-based collaborative deep model	1301 B and 644 M, LIDC	91.60	86.52	87.75	94.56	**87.13**	95.70
Alzubaidi et al*.* [4]	Gabor + SVM	500 B and 500 M, LIDC	**97**	96	-	**97**	-	-
Cai, J et al. [55]	Deep Learning	671 B and 430 M, LIDC	84.6	83.7	-	85.2	80.9	88.1
Wang et al. [56]	Deep Learning	3259 B and 5215 M, LIDC	85.23	92.79	84.56	72.89	-	92.75
Proposed model 1	Radiomics + FGSVM	324 B and 883 M, LIDC	87.6	**96.1**	88.1	64.6	85.0	89.9
Proposed model 2	Radiomics + MGSVM	324 B and 883 M, LIDC	88.0	92.7	91.1	75.4	84.9	90.9
Proposed model 3	Radiomics + CGSVM	324 B and 883 M, LIDC	87.0	91.6	90.8	74.7	83.7	91.2
Proposed model 4	Radiomics + Decision Tree	324 B and 883 M, LIDC	86.8	92.2	90.0	72.0	83.6	86.6
Proposed model 5	Radiomics + Ensemble Boosted Trees	324 B and 883 M, LIDC	87.9	91.7	91.8	77.5	84.6	92.1
Proposed model 6	Radiomics + Ensemble Bagged Trees	324 B and 883 M, LIDC	87.1	91.3	91.1	75.7	83.8	91.8
Proposed model 7	Ensemble RUSBoosted Trees	324 B and 883 M, LIDC	86.9	88.5	**93.2**	82.5	83.1	92.0
Proposed model 8	Radiomics + Ensemble Subspace Discriminant	324 B and 883 M, LIDC	86.4	93.9	88.2	65.8	83.4	91.6
Proposed model 9	Radiomics + Ensemble Subspace KNN	324 B and 883 M, LIDC	88.0	92.7	91.1	75.4	84.9	92.1

Overall highest values are marked in bold

**Table 7:** Comparison of performance metrics of proposed models with previous work when FS method is BACET

	**Methods**	**# of benign(B) and malignant nodules (M), database**	**Results (%)**					
			**Accuracy**	**Sensitivity**	**Precision**	**Specificity**	**F1-score**	**AUROC**
Dhara et al*.* [51]	2D and 3D GLCM features + SVM	279 B and 263 M, LIDC	-	89.73	-	86.36	-	95.05
Wang et al*. * [52]	Shape + intensity + Gabor + SVM	200 B and 200 M, LIDC	86	82.5	88.7	89.5	-	-
Xie et al. [53]	DCNN + Fourier + UPLBP	1324 B and 648 M, LIDC	88.28	84.15	-	90.12	-	95.70
	DCNN + Fourier + GLCM	1324 B and 648 M, LIDC	89.53	84.19	-	**92.02**	-	**96.65**
Xie et al. [54]	Multi-view knowledge-based collaborative deep model	1301 B and 644 M, LIDC	91.60	86.52	87.75	94.56	**87.13**	95.70
Alzubaidi et al*.* [4]	Gabor + SVM	500 B and 500 M, LIDC	**97**	96	-	**97**	-	-
Cai, J et al*.* [55]	Deep Learning	LIDC	84.6	83.7	-	85.2	80.9	88.1
Wang et al. [56]	Deep Learning	LIDC	85.23	92.79	84.56	72.89	-	92.75
Proposed model 10	Radiomics + FGSVM	324 B and 883 M, LIDC	81.3	**97.1**	81.1	38.3	79.1	85.7
Proposed model 11	Radiomics + MGSVM	324 B and 883 M, LIDC	85.6	90.2	90.1	72.9	82	90.4
Proposed model 12	Radiomics + CGSVM	324 B and 883 M, LIDC	84.4	91.9	87.4	63.9	81.1	90.6
Proposed model 13	Radiomics + Decision Tree	324 B and 883 M, LIDC	86.6	91.6	90.3	73.2	83.3	88.0
Proposed model 14	Radiomics + Ensemble Boosted Trees	324 B and 883 M, LIDC	87.7	91.8	91.5	76.6	84.5	92.3
Proposed model 15	Radiomics + Ensemble Bagged Trees	324 B and 883 M, LIDC	87.8	91.3	92	78.1	84.5	92.4
Proposed model 16	Ensemble RUSBoosted Trees	324 B and 883 M, LIDC	86.0	87.0	**93.4**	83.1	81.9	92.4
Proposed model 17	Radiomics + Ensemble Subspace Discriminant	324 B and 883 M, LIDC	83.5	95.7	84	50.0	80.9	90.5
Proposed model 18	Radiomics + Ensemble Subspace KNN	324 B and 883 M, LIDC	88.3	92.5	91.6	76.9	85.2	93.4

Overall highest values are marked in bold

## Conclusion and future work

With 2.21 million new cases and 1.80 million deaths, LC remains the most common and deadly type of cancer. Early detection of lung lesions and nodules is crucial in the fight against LC. Unmatched insight into the complex landscape of lung structures is offered by CT imaging. The use of CAD systems to identify lung nodules has drawn a lot of research interest in recent decades. The purpose of this study was to investigate radiomics based on geometrical, texture, and Daubechies WPT texture features for quantitative CT image analysis.

The LIDC database was used in this study. Geometrical features, texture features based on three statistical methodologies (GLCM, GLDM GLRLM), and Daubechies WPT texture features are retrieved from the nodules. Using the ensemble EFS, BOCET, and BACET, pertinent features were identified. Following a thorough evaluation process, eight features were determined to be the most significant. Lastly, various cutting-edge ML classifiers were used to classify LC as malignant or benign. The out-turn shows that, using BACET EFS, the Ensemble Subspace KNN gives best AUROC (93.4%), accuracy (88.3%) and F1-score (85.2%). FGSVM yields the best sensitivity of 97.1%. RUSBOCET gives best precision and best specificity of 93.4% and 83.1% respectively. Therefore, the methodology can be applied with efficacy to the CT based PN classification. Thus, the results can assist medical professionals in making better decisions and interventions.

Future directions include more methodical model-training modes, coalesce multimodality image features and merge multiform-omics to form ‘Medomics’. The research study can also be further extended by employing deep learning techniques braced with various optimization techniques taking into account different LC databases for earlier diagnostics, better decisions and outcomes.

## Data Availability

The dataset is publically available at 10.7937/K9/TCIA.2015.LO9QL9SX. However, upon reasonable request, the corresponding author will provide data supporting the current study's conclusions.

## Abbreviations

LC: Lung cancer
NLC: Non lung cancer
CT: Computed tomography
PN: Pulmonary nodule
ML: Machine learning
LIDC: Lung Image Data Consortium
^18^F-FDG PET/CT: Positron emission tomography with 2-deoxy-2-[fluorine-18] fluoro- D-glucose integrated with computed tomography
GLCM: Gray Level Co-occurrence Method
GLDM: Gray Level Difference Method
GLRLM: Gray Level Run Length Matrix
WPT: Wavelet Packet Transform
BOCET: Boosted classification Ensemble Trees
BACET: Bagged classification Ensemble Trees
SVM: Support vector machine
FGSVM: Fine Gaussian support vector machine
MFGSVM: Medium Gaussian Support vector machine
CFGSVM: Coarse Gaussian Support vector machine
DT: Decision Trees
RUSBOCET: Ensemble RUSBoosted Trees
FS: Feature selection
EFS: Embedded Feature Selection
WHO: World Health Organization
ROI: Region of Interest
NSCLC: Non-Small Cell Lung Cancer
TB: Tuberculosis
SPNs: Solitary pulmonary nodules
AUC: Area under the curve
KNN: K-Nearest Neighbor algorithm
RS: Random subspace
LDA: Linear Discriminant Analysis
ACOR: Autocorrelation
CON: Contrast
COR1: Correlation1
COR2: Correlation2
CP: Cluster prominence
CS: Cluster shade
DS: Dissimilarity
ENR: Energy
ENT: Entropy
HMG1: Homogeneity1
HMG2: Homogeneity2
MP: Maximum probability
SOS: Sum of Squares: Variance
SA: Sum average
SV: Sum variance
SENT: Sum entropy
DV: Difference variance
DENT: Difference Entropy
IMC1: Information Measure of Correlation1
IMC2: Information Measure of Correlation2
IDM: Inverse Difference Moment
IDMN: Inverse Difference Moment Normalized
ASM: Angular Second Moment
M: Mean
SRE: Short Run Emphasis
LRE: Long Run Emphasis
GLN: Gray Level Non-uniformity
RLN: Run Length Non-uniformity
RP: Run Percentage
LGRE: Low Gray-Level Run Emphasis
HGRE: High Gray-Level Run Emphasis
SGLGE: Short Run Low Gray-Level Emphasis
SRHGE: Short Run High Gray-Level Emphasis
LRLGE: Long Run Low Gray-Level Emphasis
LRHGE: Long Run High Gray-Level Emphasis
